# Some Notes on the Treatment of Puerperal Septic Disease

**Published:** 1910-10-22

**Authors:** Knyvett Gordon

**Affiliations:** formerly Medical Superintendent of Monsall Hospital and Lecturer on Infectious Diseases in the University of Manchester.


### Hospital Clinics.

### SOME NOTES ON THE TREATMENT OF PUERPERAL SEPTIC DISEASE.

By KNYVETT GORDON, M.B. (Cantab.), formerly Medical Superintendent of Monsall Hospital and Lecturer on Infectious Diseases in the University of Manchester.

In the following notes I propose to give some conclusions to which a personal experience of some 250 cases of the more severe types of puerperal infection has led me; the patients were admitted to Monsall Hospital, and the bacteriology of the cases was studied—as far as it was possible to do so without inoculation experiments—in the laboratory attached to that institution.

### Puerperal Fever in Private Practice.

As a matter of fact, the disease which, for want of a better name, is known as puerperal fever is at present in rather an unfortunate position as far as the practitioner is concerned. Of recent years the literature on the subject has been voluminous, and the discussions about its pathology by learned societies have been both frequent and acrimonious; but the patients themselves are not usually accessible for teaching purposes, and the practising physician, who as a rule is not particularly interested in descriptions of the symptoms which a bacteriologist thinks the patient ought to have, is in consequence somewhat nonplussed when he meets with a case in his own practice.

### A Note on Pathology.

I shall not now discuss the pathology of the disease, but it is necessary to point out—as it gives the keynote to adequate treatment of the patients—that puerperal sepsis is due to a definite endometritis, which is caused in the vast majority of cases by infection of the uterus with streptococci or bacillus coli or both combined. It is true that infection from vaginal tears or perineal lacerations may give rise to transient rises of temperature with consequent malaise, but unless this reaches the uterus also, serious disease does not, as a rule, result.

When endometritis has occurred, the infection may travel along one or more of three routes, and it may progress either slowly or quickly. Thus, the organisms may reach the circulating blood, either with such rapidity as to produce death from general septicæmia in two or three days—perhaps even sooner—or there may be some attempt at defence, and thrombosis of the veins in and around the uterus may occur, with pyæmia as the result. Or the path may be *viâ* the lymphatics; the process is then usually slow, and parametritis, with or without suppuration, is set up. I have, however, seen a few cases in which an inflammation of the connective tissue of a pseudo-gangrenous type resulted with a rapidly fatal issue. Lastly, the organisms may travel along the Fallopian tubes, and cause either rapid general peritonitis, or, if the progress is slower, adhesions, pelvic peritonitis, pyosalpinx, and so on.

### How Pathology affects Treatment.

On the vexed question as to how the organisms reach, or are placed in, the uterus in the first instance, I shall not touch. When we leave the pathology of the disease and come to questions of treatment, we are faced with a great, and at present, insuperable difficulty, namely, that we have to consider not only the nature of the disease but the extent to which the patient is reacting to the invading organisms. Unfortunately we have, as yet, no method available at the bedside by which we can measure the resistance of the host, and that is what we want very badly. In fact, though careful examinations were made of the blood—in which direction it would seem to be most likely that help would be forthcoming—the more one saw of the patients themselves the less did one rely on such determinations, and the more did one lean on such "indefinite" signs as the state of the pulse, tongue, temperature, and so forth, not omitting the aspect of the patient. I would not for one moment, however, be thought to deride the *rôle* of the clinical pathologist in the study of puerperal fever, for I am convinced that light must come by further research, but it seems necessary to utter a word of caution against accepting obviously unfinished work in this direction as authoritative.

### How to Deal with the Case.

Coming now to the patient herself, we have four tasks before us: to deal with the endometritis (in order to prevent as far as possible any further dissemination of infective matters); to assist in the elimination of the products already formed; to combat the anatomical results of the extension of the disease along the aforesaid paths; and to strengthen the general resistance of the patient.

Perhaps the first is the most important; it is certainly the most difficult. In the milder forms of the disease it is probable that between the organisms and the roads into the interior of the host is a barricade of leucocytes, which is possibly to a certain extent efficacious, though it is hardly as sacrosanct as some pathologists would have us believe. It is not, however, desirable to do anything at this stage which will damage it if this can be avoided. So we endeavour to attack the organisms alone, and here there are two methods available, to douche the cavity of the uterus or to swab the endometrium itself. Now, there are two very different ways of procedure with the douche: we can either place the patient in the lithotomy position, insert a weighted speculum, and draw down (and thus open) the cervix with volsellæ, or we can conduct the operation with the patient on her side in bed and introduce the canula without exposure, by the sense of touch alone, the cervical canal in this case remaining more or less closed. For the latter, and undoubtedly most common, method I have nothing but condemnation, in that there must be more or less obstruction to the return of the fluid. The first procedure is better, but there is still the objection that all that is really done is to flush out the interior of the uterus, and we undoubtedly want to do more than this—namely, to attack (if it be possible) the organisms just inside the endometrium itself. Moreover, when we have thus thoroughly exposed our patient we may just as easily adopt the undoubtedly more efficacious method of swabbing. For this purpose I have generally used undiluted izal, but the name of local antiseptics is legion. After the swabbing, which must be thorough, I have been accustomed to pack the uterus and vagina with a gauze tampon wrung out of a mixture of equal parts of glycerine and the selected antiseptic solution, which is removed after twenty-four hours and not reinserted unless the whole process has to be repeated. In the milder cases this method is undoubtedly useful, but the perennial difficulty meets us here again in that we do not know what is a mild case, or, in pathological language, whether the barrier of leucocytes has not already been broken down. Inasmuch as it is invariably found to be absent in the fatal cases, it is obvious that it is inadvisable to assume that it is present, or effective, when the patient is evidently ill, and to withhold more active local measures on the ground that they may damage it.

### Some Statistics.

On this point, however, it is possible to give some details. Out of the first seventy-nine cases in which general methods of treatment (including serum) were employed, with or without intra-uterine douches, thirty-seven died—a mortality of 46 per cent. In the next eighty-six cases, where the sharp curette was used (followed by swabbing without douching) the mortality fell to 23 per cent., whilst it was lower still in the remaining cases of the series.

I feel confident, therefore, that in the more severe cases it is best to expose the uterus thoroughly and to use a large but light, sharp curette, followed by a thorough swabbing with the selected antiseptic and by subsequent packing of the uterine and vaginal cavities. Incidentally, in these severe cases the uterine cavity is usually over six inches in length, and it is not easy to see how its interior can be adequately treated by the finger alone, as is so often advised.

### Subcutaneous Saline Infusions.

Then it is desirable to assist in the elimination of toxins as far as we can, and here saline subcutaneous infusions are very useful; perhaps the most convenient method is to allow two pints of normal saline solution to run into the axilla daily; the process takes about half an hour. Then calomel, in grain or half-grain doses, given every hour until from three to six grains have been administered, is a very useful drug. I must admit that in the use of antistreptococcic serum I have been rather disappointed, though I have given it freely in doses of 100 c.c. or more, and have used many different varieties. Still there is no reason why it should not be used in any given case, as it does no harm unless the patient has had serum before, when the dangers due to anaphylaxis must be borne in mind.

### Surgical Treatment.

We now come to the surgery of puerperal sepsis, and here again the problem is a complex one, for we obviously must do as little as possible on account of the weakening of the resistance of the host which has already taken place. Still, we can do something, and in practice we have to consider three conditions—first, general peritonitis, then intense pelvic peritonitis (which may or may not be merging into the former condition), and lastly, the indications for hysterectomy. I do not include the opening of obvious pelvic abscesses, for the necessity of which there can be no doubt. The procedure for this purpose can be carried out per vaginam without the necessity for a general anæsthetic.

### The Advantages of Local Anæsthesia.

There can nowadays be no doubt that we must open the abdomen in cases of obvious general peritonitis, and I think that the view is now generally held that we should not as a rule wash out the cavity; we drain as freely as possible and in the most dependent parts, and subsequently prop the patient up in the sitting position and give saline freely, subcutaneously or per rectum. But I do not think that the importance of dispensing with a general anæsthetic is sufficiently recognised. In point of fact, a dose of alcohol one hour before the operation, followed by a hypodermic injection of morphia half an hour later, with infiltration of the abdominal wall with eucaine or other local anæsthetic is usually sufficient, and, looking back on some previous cases, I am inclined to think that the general anæsthetic may have turned the scale against the patient.

### Peritonitic Cases.

The existence of intense peritonitis, which is apparently limited to the pelvis, presents a much greater difficulty. Obviously our impression is that the abdomen should not be opened, but that the patient should rather be left until the acute stage is over and the collections of pus and the results of adhesions then dealt with by abdominal section or by the vaginal route, and in the great majority of cases this is undoubtedly the best course to pursue. For one thing, if one does open the abdomen the technical difficulties are great. I have vivid recollections of attempts to deal with peritoneum that looked like red flannel, and of the numerous ligatures and sutures that cut through without effecting hæmostasis. Still, there are undoubtedly cases in which, if we only knew it, it is best to open the abdomen in the acute stage and remove a pyosalpinx, or possibly the uterus itself, and drain the pelvic peritoneum. Certainly, I can recall cases in which I saw pelvic peritonitis go unrelieved and appear on the post-mortem table in a stage at which the possibilities of a successful laparotomy looked greater than they had done during life. Mere drainage can be effected by a posterior colpotomy, and I have sometimes found this useful, but there is a disadvantage in these acute cases, that if anything does bleed, effective hæmostasis is very difficult indeed through a vaginal wound, and they are apt to end in a hurried laparotomy.

Hysterectomy Cases.

The last point is, what cases, if any, are suitable for hysterectomy? Here my own experience has been but small—seven cases with one recovery—because I have always felt that we should try local disinfection first, and on the whole I am still of that opinion. Looking back, however, on the whole series, one cannot but feel that one has lost cases through not removing the uterus, and I should be now inclined to say that when the uterine wall is soft and friable with a putrid discharge, while the blood does not contain streptococci, and there is a leucocytosis, it would be better to lose no time in removing the uterus, and I have had one successful case on these lines. The type in which I believe hysterectomy to be contraindicated is where the uterus is smooth and the blood contains organisms.

The indications for supporting the patient's strength do not differ from those seen in other acute pyrexial illnesses, and need not be detailed here. Obviously the sheet-anchor is careful feeding by a skilled nurse.

